# Layer contribution to optical signals of van der Waals heterostructures†

**DOI:** 10.1039/d0na00906g

**Published:** 2021-04-10

**Authors:** Su-Yun Wang, Guo-Xing Chen, Qin-Qin Guo, Kai-Xuan Huang, Xi-Lin Zhang, Xiao-Qing Yan, Zhi-Bo Liu, Jian-Guo Tian

**Affiliations:** The Key Laboratory of Weak Light Nonlinear Photonics, Ministry of Education, School of Physics and Teda Applied Physics Institute, Nankai University Tianjin 300071 China liuzb@nankai.edu.cn; Renewable Energy Conversion and Storage Center, Nankai University Tianjin 300071 China; The Collaborative Innovation Center of Extreme Optics, Shanxi University Taiyuan Shanxi 030006 China

## Abstract

The optical signals (such as Raman scattering, absorption, reflection) of van der Waals heterostructures (vdWHs) are very important for structural analysis and the application of optoelectronic devices. However, there is still a lack of research on the effect of each layer of two-dimensional materials on the optical signals of vdWHs. Here, we investigated the contribution from different layers to the optical signal of vdWHs by using angle-resolved polarized Raman spectroscopy (ARPRS) and angle-dependent reflection spectroscopy. A suitable theoretical model for the optical signal of vdWHs generated by different layers was developed, and vdWHs stacked by different two-dimensional (2D) materials were analyzed. The results revealed a strong dependence of the relative strengths of the optical signals of the upper and lower layers on the thicknesses of 2D materials and the SiO_2_ layer on the Si/SiO_2_ substrate. Interestingly, on the 285 nm SiO_2_/Si substrate, the contribution to the optical signal by the underlying 2D material was much greater than that by the upper layer. Furthermore, optical signals originating from different layers of twisted black phosphorus (BP) for different twist angles were studied. There is great significance for optical spectroscopy to study vdWHs, as well as the development of better twisted 2D materials and moiré physics.

## Abstract 2

The contribution from the upper and lower layers to the optical signal of vdWHs is dependent on the thicknesses of 2D materials and substrates.

## Graphical abstract

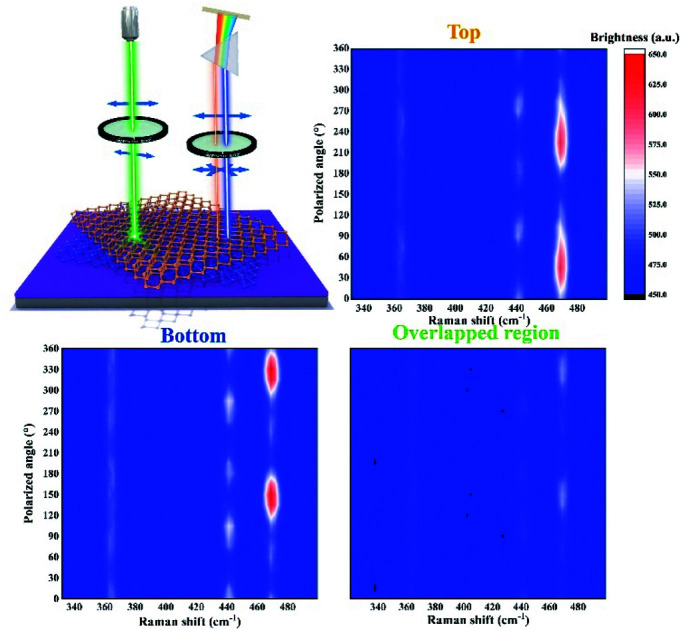

## 1. Introduction

van der Waals heterostructures (vdWHs) have received increasing attention due to their unique electronic, optical and mechanical properties,^1–8^ and have shown numerous interesting physical behaviors, such as Hofstadter's spectra,^1^ nonlinear optics,^2^ moiré excitons,^3^ topological polaritons,^4–6^ and unconventional superconductivity.^7,8^ These features have great application potential in electronics and photonics.

The optical properties of two-dimensional (2D) materials are worthy to be studied to help to reveal the intrinsic physics of strong light–matter interactions,^9,10^ and may contribute to designing smart optoelectronic devices in the field of nanotechnology.^11–13^ Nowadays, most current studies extend from the optical properties of independent 2D materials toward those of vdWHs.^14–17^ However, since the optical signal in vdWHs depends not only on the dielectric constant and thickness of each layer, but also on the stacked structure, the contribution of each layer to the optical signal is very complicated. At present, systematic studies related to optical signals from different layers of heterojunctions are still lacking. Therefore, the analysis of optical signals in vdWHs is of great significance to the characterization of structures and the application of optoelectronic devices, especially to study the relative contribution of each layer to optical signals in vdWHs.

Here, we used angle-resolved polarized Raman spectroscopy (ARPRS)^18–21^ and the anisotropic imaging technique (AIT)^22^ to characterize the optical signal generated by different layers, which was widely applied to determine the crystalline orientation of layered 2D anisotropic materials. At the macro-scale, the superimposition of two layers of the same medium (top and bottom layers) revealed the optical signal to come only from the top medium. However, at the micro-scale, the optical signal coming from the bottom black phosphorus (BP) layer appeared when the top BP layer almost vanished due to stacking of two BP flakes. The physical phenomenon was attributed to stacking of two or three BP samples together, which was also present in other vdWHs like BP/ReS_2_. Based on this phenomenon, a theory based on multiple interference was then developed to describe the relative Raman intensity of each mode in vdWHs. The theoretical and experimental data were used to identify the relationships between the thicknesses of vdWHs and SiO_2_ layers as a function of the relative Raman intensity of BP/BP, graphene (G)/BP and BP/ReS_2_ vdWHs, respectively. We also revealed how the stacking angle affected the Raman intensity of BP/BP vdWHs. The experimental data also showed that the reflection spectroscopy signal generated by different layers is a function of the thicknesses of vdWHs and SiO_2_ layers. Overall, these findings would surely benefit future studies of optics, optoelectronics, and moiré physics in vdWHs.

## 2. Theoretical model of Raman intensity in vdWHs

ARPRS was measured using a laser with linear polarization, and the scattered light was measured using a linear polarizer to independently measure components parallel to the incident laser polarization. All the experimental and theoretical results were obtained under the parallel configuration. The corresponding intrinsic Raman intensity for a given mode could be calculated according to eqn (1):^18–20^1
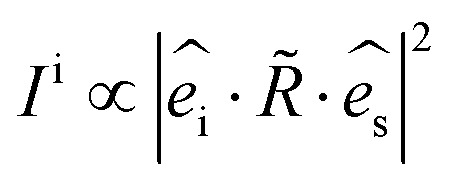
where 
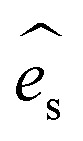
 and 
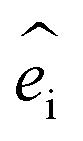
 are the polarization vectors of the scattered and incident photons, respectively.

However, eqn (1) would not be enough to describe the Raman intensity coming from the sample, that is because the essential interference factor resulting from the interference effects in laser and Raman scattered lights is not taken into account.

For two superimposed layers of 2D materials, the Raman intensity of the overlapped region can be expressed according to eqn (2):

2 *I*_ove_ = *I*_top_ + *I*_bot_

where *I*_ove_ is the Raman intensity of the overlapped region. *I*_top_ represents the Raman signal coming from the top 2D material layer, and *I*_bot_ denotes the Raman signal induced by the bottom 2D material layer. The Raman intensity can also be expressed following eqn (3):^23,24^

3 *I*_top_ ∝ *F*_top_*I*^i^_top_, *I*_bot_ ∝ *F*_bot_*I*^i^_bot_, *I*_ove_ ∝ *F*_top_*I*^i^_top_ + *F*_bot_*I*^i^_bot_

where *F*_top_ and *F*_bot_ are interference factors of the top and bottom 2D material layers. For overlapped regions consisting of two different 2D materials, the Raman signal of each mode would come from either the top or the bottom 2D material layers.

The direction of the maximum Raman intensity was used to decide the crystalline orientation of 2D materials,^18^ and interference factors along this direction were mainly discussed. The parameter *m*, which is the ratio of the maximum Raman intensity of the independent to overlapped region, can be expressed by eqn (4):4
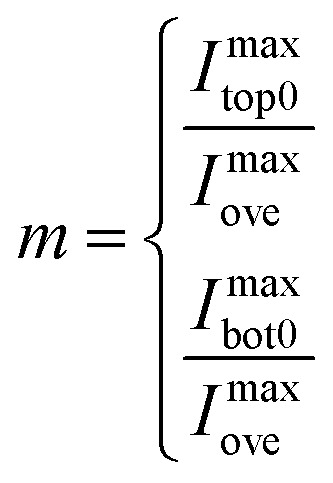
where *I*^max^_top0_ and *I*^max^_bot0_ are the maximum Raman intensity of independent top and bottom 2D materials. For 0 < *m* < 1, the maximum Raman signal of the overlapped region is larger than that of the independent region, while for *m* > 1, the situation is the opposite.

For van der Waals homo-structures, the Raman signals of the overlapped region would be generated from both the top and bottom layers. Thus, the values of *s* would be used to describe the Raman signal contributions of both the top and bottom layers (eqn (5)):5
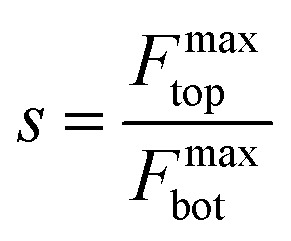


The obtained physical phenomena were then translated into mathematical models. For *s* < 1, the maximum interference factor of the bottom layer was larger than that of the top layer. Therefore, the maximum Raman signal coming from the bottom layer was larger than that originating from the top layer. For *s* > 1, the maximum Raman signal induced by the top layer was superior to that generated by the bottom layer.

BP/BP van der Waals homo-structures were utilized to verify the proposed theory. To this end, ARPRS was carried out on the bottom BP, top BP and overlapped region. A^1^_g_, B_2g_ and A^2^_g_ modes were considered as the three characteristic Raman modes of BP. The Raman tensor of A_g_ and B_2g_ modes in the backscattering geometry in complex forms can be expressed according to eqn (6) and (7):^25^6
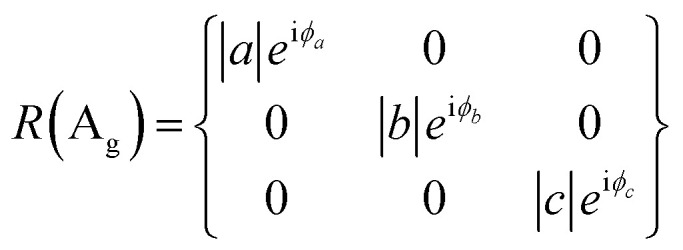
7
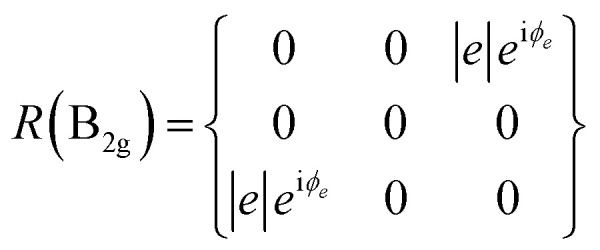


The corresponding Raman intensity under the parallel configuration for a given mode would be proportional to eqn (1), and the polarization vectors with parallel polarization can be expressed following eqn (8):^20^8
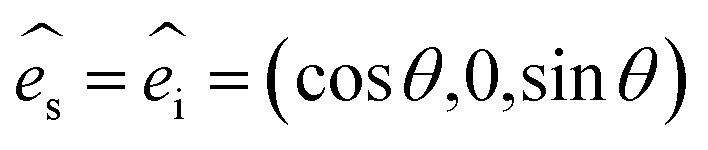
where *θ* represents the angle between the laser polarization and zigzag (ZZ) direction of BP flakes.

The Raman intensity of A_g_ modes under the parallel configuration might be given by eqn (9):^20^9
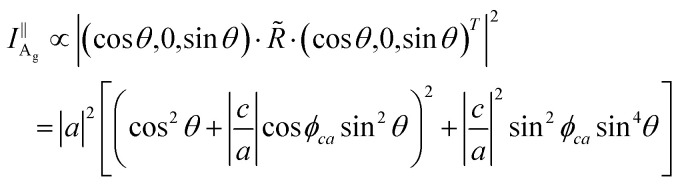
where *ϕ*_*ca*_ is the phase difference between the Raman tensor elements *c* and *a*.

Besides, the Raman intensity of the B_2g_ mode under the parallel configuration can be provided by eqn (10):^20^10
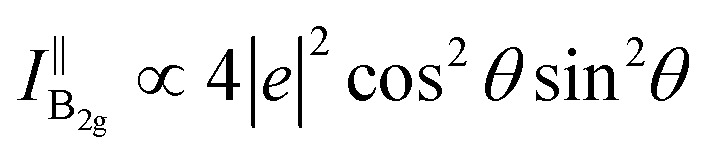


The crystalline orientation of BP can be clearly distinguished in eqn (9). Thus, eqn (9) was used to fit the experimental data of BP. The values of *a*, *c*, *e*, and *ϕ*_*ca*_ have been reported in the literature,^20^ so *I*^i^ can be calculated by eqn (9). Note that the Raman signal of the overlapped region consisted of those coming from the bottom and top BP, and the relative proportion can be represented by the parameter *s*. The parameter *m* is the ratio of maximum Raman intensity of the independent BP to the overlapped region. The parameters *m* and *s* are the main contents we will discuss.

Next, interference factors were introduced into the calculations. Unlike previous studies, the physical model of the experiments consisted of five phases instead of four, and BP was considered as an anisotropic instead of isotropic 2D material. Like previous studies, the proposed theory could also be developed using multiple reflection interference. On the other hand, the Raman intensity can be separated into two parts: (i) the Raman signal induced by the bottom BP layer and (ii) the Raman signal generated by the top BP layer. Thus, Raman signals generated from the bottom and top BP layers can be calculated independently. Besides, an approximation was introduced before any calculation of intensities. The approximation assumed the variation of *n* along the *p*–*s* plane as an elliptical shape, where *n* along the *p* direction is denoted as *n*_*p*_, and *n* along the *s* direction is called *n*_*s*_. *n*_ZZ_ and *n*_AC_ are the refractive indexes of BP along with ZZ and armchair (AC) directions. Note that *n*_ZZ_ and *n*_AC_ represent the two axes of the ellipse. Therefore, *n*(*θ*) along a given direction *θ* (equivalent to the polarized light direction) can be expressed according to eqn (11):^26^11
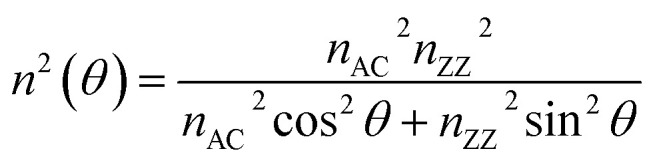
*n*(*θ*) represents the refractive index of the top BP layer along the given direction *θ*. *Γ* is the twist angle, and *n*(*Γ* + *θ*) denotes the refractive index of the bottom BP. This approximation greatly reduced the calculation load. For thin BP layers, the change in the polarization angle was smaller. In other words, thinner BP layers yielded more precise approximations.

**Fig. 1:**
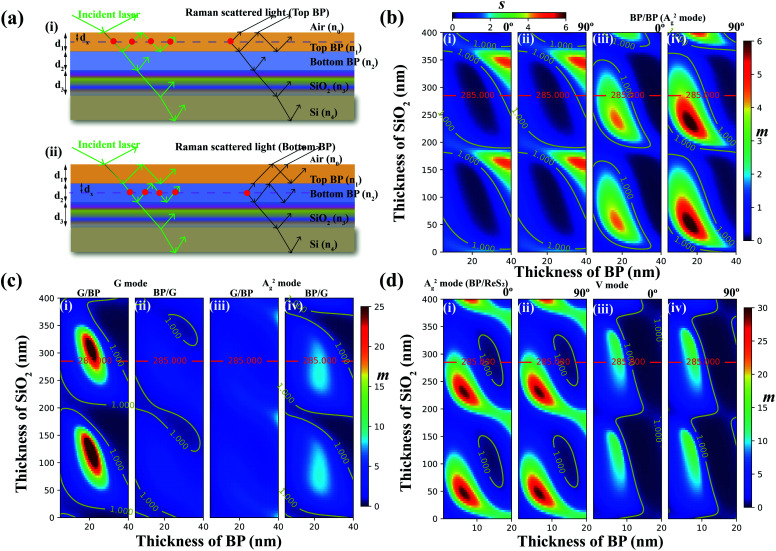
The calculation of interference factors of the Raman signal for vdWHs under the parallel configuration. (a) Scheme of five phases of air/top BP/bottom BP/SiO_2_/Si. The Raman signal of the overlapped region generated by (i) top BP and (ii) bottom BP. (b) The variations in *s* and *m* as a function of thicknesses of SiO_2_ and BP layers. The value of *s* was calculated and the stacking angle was (i) 0° and (ii) 90°. The value of *m* was calculated and the stacking angle was (iii) 0° and (iv) 90°. (c) The variation in *m* as a function of thicknesses of SiO_2_, G, and BP layers. (i) The heterostructure was made of top G and bottom BP for the G mode. (ii) The heterostructure was made of top BP and bottom G for the G mode. (iii) The heterostructures were made of top G and bottom BP for the A^2^_g_ mode. (iv) The heterostructure was made of top BP and bottom G for the A^2^_g_ mode. The laser polarization plane was along the AC direction. (d) The calculation result of *m* as a function of thicknesses of SiO_2_, BP and ReS_2_. BP is the top layer. (i) G mode of BP. The stacking angle between the ZZ direction of BP and the *b*-axis of ReS_2_ is 0°. (ii) A^2^_g_ mode of BP. The stacking angle is 90°. (iii) V mode of ReS_2_. The stacking angle is 0°. (iv) V mode of ReS_2_. The stacking angle is 90°.

For the bottom BP layer (Fig. 1(a)(i)), the net absorption at position *x* can be defined as *F*^bot^_ab_(*x*). The factors related to reflection of the Raman signal at position *x* can be defined as *F*^bot^_sc_(*x*) (see ESI note 1 for more calculation details†). The whole interference factors can be recalculated when considering both terms. Since the Raman intensity would be proportional to the enhancement factors (*F*^bot^_ab_(*x*) and *F*^bot^_sc_(*x*)), the total interference factors caused by the multiple interference effect could be given by eqn (12):^19,20^12
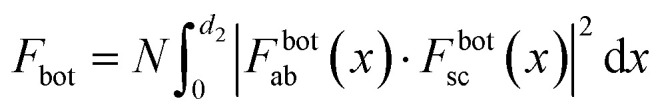
where *N* is the normalization factor.

For the top BP layer shown in Fig. 1(a)(ii), the total interference factors can also be expressed by eqn (13):^19,20^13
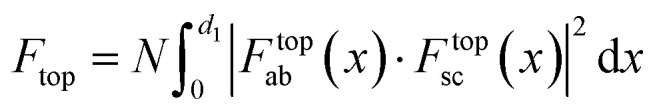


The analysis of the above-mentioned considerations revealed proportional relationships between the Raman intensities of the bottom or top BP layers and interference factors *F*. Note that the refractive index of BP reported in the literature was used for calculations.^27^

**Fig. 2:**
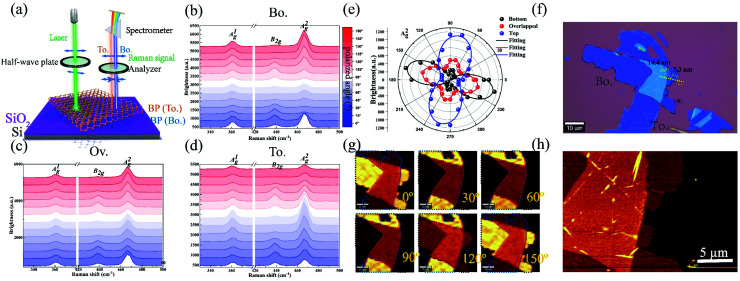
Polarization dependence of Raman modes for BP under the parallel configuration on a 285 nm SiO_2_/Si substrate with a 532 nm excitation wavelength. (a) The schematic diagram of the polarization dependence measurement of Raman modes for top BP, bottom BP, and the overlapped region. The green light represents the laser source with a wavelength of 532 nm. The blue light and the orange light represent the Raman signal generated by the bottom and top BP in the overlapped region. (b–d) The Raman spectra of bottom BP, top BP, and the overlapped region at different polarization angles. (e) A^2^_g_ mode as a function of the polarization angle for bottom BP, top BP, and the overlapped region. (f) The optical image of the BP/BP junction. (g) The Raman mapping for the sample at 0°, 30°, 60°, 90°, 120°, and 150° polarization angles. (h) The AFM image of the sample. The thicknesses of the bottom BP and the overlapped region are 7.3 and 14.4 nm.

The variations in *m* and *s* as a function of thicknesses of SiO_2_ and bottom BP layers for the A^2^_g_ mode are provided in Fig. 1(b), note that thicknesses of the top and bottom BP layers were kept the same so that the thickness of the overlapped region would be twice that of the bottom or top BP layers. Also, the theoretical excitation wavelength of 532 was employed in the simulation. In Fig. 1(b), the zone of ratio *s* < 1 can be visualized from the picture, which is wider than that of *s* > 1, indicating that the maximum Raman signal induced by the bottom BP layer was larger than that generated by the top BP layer (Fig. 1(b)(i and ii)). This phenomenon predicted by the theory was abnormal but firstly observed by experiments (Fig. 2(g)). On the other hand, the zone of *s* < 1 looked much larger for the Si substrate with a 285 nm SiO_2_ layer. The Si substrate with a 285 nm SiO_2_ layer was often used in the experiment because of the high contrast of 2D materials.^28^ The value of *m* increased first and then decreased as the BP thickness deposited on a 285 nm thick SiO_2_ layer rose from 5 nm to 40 nm. In Fig. 1(b)(iii and iv), the zone of *m* < 1 changed periodically with the thickness of the SiO_2_ layer and twist angle, but the latter demonstrated a smaller influence than the former. The theoretical excitation wavelength of 514.5 nm was also employed in the simulation (Fig. S3†).

Moreover, the hetero-structures formed by anisotropic and isotropic 2D materials, BP and graphene were also studied, and the simulation results are displayed in Fig. 1(c)(i–iv). For the zone of *m* < 1, the Raman signal of the overlapped region was much larger than that of the independent region. This suggested an enhancement in the Raman signal when 2D materials were stacked together. The experimental results are demonstrated in Fig. 4(e and f) showing that *m* > 1 on the Si substrate with a 285 nm SiO_2_ layer.

**Fig. 3:**
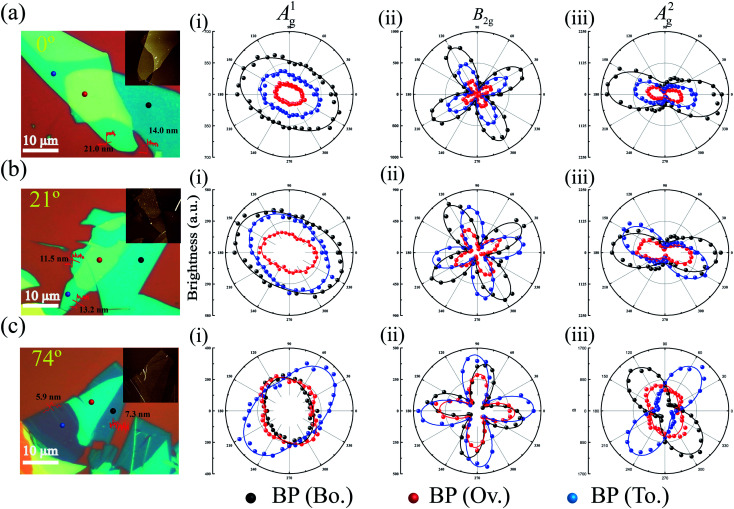
Polarization dependence of A^1^_g_, B_2g_ and A^2^_g_ modes for different stacking angles on a 285 nm SiO_2_/Si substrate with a 514.5 nm excitation wavelength. (a) The optical image of the BP heterojunction for a 0° stacking angle. Inset is the AFM image. (i) A^1^_g_, (ii) B_2g_ and (iii) A^2^_g_ modes as a function of the polarization angle. (b) The optical image of the BP heterojunction for a 21° stacking angle. Inset is the AFM image. (i) A^1^_g_, (ii) B_2g_ and (iii) A^2^_g_ modes as a function of the polarization angle. (c) The optical image of the BP heterojunction for a 74° stacking angle. Inset is the AFM image. (i) A^1^_g_, (ii) B_2g_ and (iii) A^2^_g_ modes as a function of the polarization angle.

**Fig. 4:**
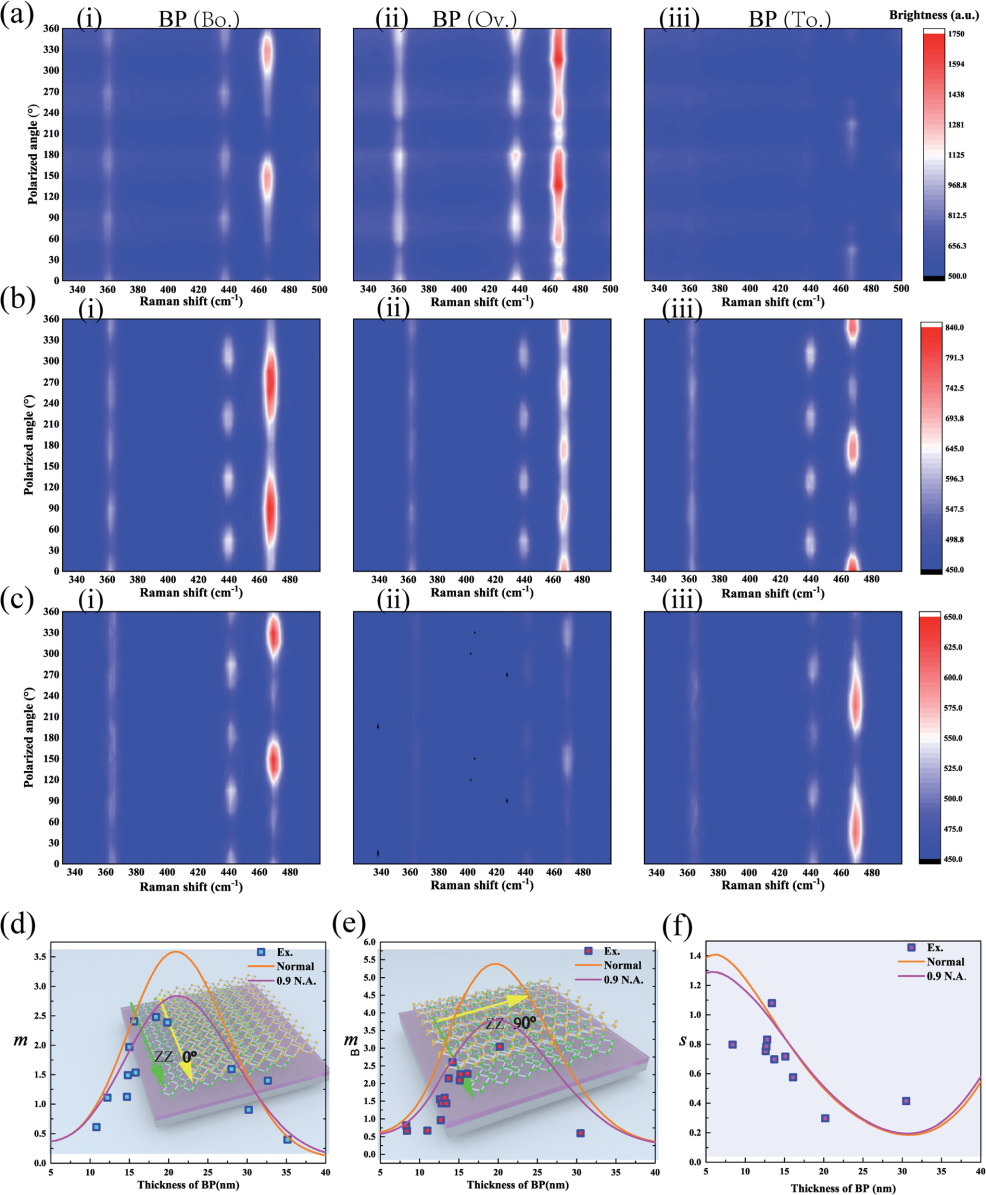
Polarization diagram of the Raman intensity of the A^2^_g_ mode for the BP/BP junction on the Si/SiO_2_ substrate under the parallel configuration with a 532 nm excitation wavelength. (a–c) The polarization-resolved Raman spectra of the BP/BP junction on the Si substrates with 0, 30, and 100 nm SiO_2_ layers as a function of the polarization angle. The Raman signal was generated by (i) bottom BP, (ii) overlapped region, and (iii) top BP. (d and e) The theoretical and experimental data of the value of *m* (the ratio of the Raman intensity of bottom BP to that of the overlapped region) as a function of the thickness of the BP/BP junction on the 30 nm SiO_2_/Si substrate. (f) The theoretical and experimental data of the value of *s* (the maximum interference factors of bottom BP to that of the top BP) as a function of the thickness of the BP/BP junction on the 30 nm SiO_2_/Si substrate.

The variations in *m* as a function of the thicknesses of SiO_2_, BP, and ReS_2_ layers for A^2^_g_ mode are illustrated in Fig. 1(d)(i and ii). Note that the thicknesses of the top BP and bottom ReS_2_ layers were kept the same so that the thickness of the overlapped region would be twice that of the top BP layer. For the top layer made of BP (Fig. 1(d)(i and ii)), the zone of *m* > 1 changed periodically with the thickness of the SiO_2_ layer and twist angle, but the latter demonstrated a smaller influence than the former. Fig. 1(d)(iii and iv) show the variation in *m* as a function of the thicknesses of SiO_2_ and ReS_2_ layers for the V mode of the ReS_2_ layer. We found that results shown in Fig. 1(d)(iii and iv) look similar to those of Fig. 1(d)(i and ii), but with a larger zone of *m* < 1, and the values of *m* on the 285 nm SiO_2_ layer were smaller than those in Fig. 1(a)(i and ii).

## 3. Experimental methods

Few layers BP, ReS_2_ and G were exfoliated using a mechanical method^29,30^ from the bulk crystal and then deposited on the SiO_2_/Si substrate and polydimethylsiloxane (PDMS) substrate. For vdWHs, the dry-transfer technique was used to transfer the top layer on the PDMS substrate onto the top of the bottom layer on the SiO_2_/Si substrate. The vdWHs are fabricated successfully. To make sure that the thicknesses of bottom BP and top BP are the same, BP was prepared on the PDMS substrate using a mechanical method, the BP was transferred onto the top of the graphene, the BP flake was separated into two pieces,^31^ one of the two pieces were rotated manually by a twist angle, two pieces were stacked together and twisted black phosphorus (TBP) was fabricated successfully.

The thickness of BP is determined by atomic force microscopy (AFM). For the Raman characterization, the WITec alpha300 RAS system with the 532 nm excitation laser and the RENISHAW RM2000 system with the 514.5 nm excitation laser were used to obtained the Raman spectrum. The sample was excited under the 514.5 nm laser at a power of 1 mW under a 50× objective lens with a N.A of 0.65. The other sample that was used to prove the theory was excited under the 532 nm laser at a power of 0.1 mw under a 100× objective lens with a N.A of 0.9. Under the parallel configuration, the polarization directions of the excitation laser and scattered light were rotated to obtain the polarized-Raman spectrum with increasing rotation angle in steps of 15 and the sample was fixed. For the AIT measurement, a Nikon optical microscope (Eclipse Ci-S) with a fiber optic illuminator (Halogen lamp, 12 V, 150 W, LS-LHA) was used. Angle-dependent reflectance spectra were obtained using a CCD detector (YW500 Camera) when the polarizer was rotated. 10 nm band-pass filters were used to generate quasi-monochromatic light (Thorlabs, FB600-10). 40×/0.65 dry objective lenses were used.

## 4. Results and discussion

The polarization Raman technique was carried out on the bottom, top BP, and overlapped region under the parallel configuration (see ESI note 12† for the measurement under the cross and unpolarized configurations). The preparation of vdWHs is provided in the Methods section. Fig. 2(a) gathers the polarization dependence measurements of Raman modes under the parallel configuration. The Raman signal induced by bottom BP (orange light) is larger than that generated by top BP (blue light). The three characteristic Raman modes A^1^_g_, B_2g_, and A^2^_g_ of BP are shown in Fig. 2(b and d). The images obtained by the optical microscopy and AFM techniques for the bottom and top BP layers and the overlapped region are provided in Fig. 2(f and h). The evolution of Raman spectra with the rotation of polarized light about the surface normal of the BP/BP junction is presented in Fig. 2(b and d). The peak positions remained unchanged, but the intensity of each peak varied strongly with the polarization angle. Thus, the A^2^_g_ mode could be used to identify the crystalline orientation of BP.^18–21^

The polarization dependence of the Raman intensity for the A^2^_g_ mode is depicted in Fig. 2(e). The twist angle was set to 90° and the azimuth angle of maximum Raman intensity of the overlapped region was parallel to the crystalline orientation of bottom BP. The polarization dependence of the A^2^_g_ mode for the overlapped region revealed strong anisotropy properties.^32^ The overlapped region could be regarded as a synthetic anisotropic independent entity. Considering the polarization angle along the maximum Raman intensity direction as crystalline orientation of the BP layer and overlapped region,^18^ we could easily find that the crystalline orientation of the overlapped region (CRO) is parallel to that of bottom BP instead of top BP. Such a phenomenon can also be visualized from Raman mapping (Fig. 2(g)), where the color variation tendency of the overlapped region was consistent with that of the bottom BP layer. That is because the interference factors of bottom BP were larger than those of top BP, and the anisotropic properties of the overlapped region mainly depended on bottom BP.

Unique Raman behaviors could also exist at different stacking angles, layers (Fig. S8†), and different Raman active modes. The polarization dependence of A^1^_g_, B_2g_, and A^2^_g_ modes under the parallel configuration at different stacking angles is exhibited in Fig. 3. At a stacking angle of 0°, the crystalline orientation of the overlapped region for the A^2^_g_ mode was the same as those of the bottom and top BP. The Raman intensities were weaker than those of bottom and top BP in all cases studied in Fig. 3. This is because the parameter *m* is always bigger than 1 in the thickness range of BP we studied. The crystalline orientation obviously skewed toward the crystalline orientation of bottom BP, which was found in all Raman active modes.

The thickness of the SiO_2_ layer could impact the interference factors based on multiple interference.^20,23,24^ To clarify the reason why the interference factors of bottom BP were larger than that of top BP, BP/BP junctions assembled on the Si substrate with different thicknesses of SiO_2_ layers were tested under the parallel configuration. The reason can be extracted by looking at the variation in the Raman intensity of the A^2^_g_ mode under different thicknesses of the SiO_2_ layer. For the 30 nm SiO_2_/Si substrate, the interference factor of bottom BP was the same as that of top BP (Fig. 4(b)), which led to the crystalline orientation of the overlapped region parallel to that of bottom and top BP (*s* ≃ 1). Fig. S1† shows that the crystalline orientation of the overlapped region skewed toward that of the top BP (*s* > 1). At fused Si and 100 nm SiO_2_/Si substrates, the crystalline orientation of the overlapped region depended on bottom BP (Fig. 4(a)). Consequently, the thickness of the SiO_2_ layer was one reason to consider since it can strongly influence the interference effect. Thus, multiple interference may explain the behavior of crystalline orientation dependence of the overlapped region.

The measurement of *m* and *s* for the A^2^_g_ mode was utilized to prove the correctness of the proposed theory. The polarization Raman technique was carried out on the BP/BP junction under the parallel configuration. The thicknesses of bottom and top BP were kept the same, so that the thickness of the overlapped region was twice that of bottom BP or top BP. The Raman intensity for the A^2^_g_ mode of the bottom BP was almost the same as that of the top BP along AC and ZZ directions (Fig. S2†). The ratio *m* of the Raman intensity was then calculated for bottom BP and TBP as a function of thicknesses of the independent BP and the SiO_2_ layers. The SiO_2_ layer with a thickness of 30 nm was employed to test the variation of Raman intensity with the thickness of BP. In our analysis above, the normal incidence is assumed. However, the numerical aperture (N.A.) of the objective lens is large, and it needs to be considered. The simulation results of normal incidence and 0.9 N.A. were given respectively. The values of *m* decreased slightly as the N.A. increased, and that of *s* almost kept the same (Fig. 4(d and e)). The objective lens with 0.9 N.A. was used in the measurement, and the experimental data agreed well with the theory (Fig. 4(d and e)). The region where the Raman intensity of the overlapped region was smaller than that of the independent region can be directly visualized [Fig. 1(b)(iii and iv)]. Due to the fact that the interference factor for the 90° BP/BP junction between the ZZ and AC directions (*F*_ZZ_/*F*_AC_) was ∼1.03 (see ESI note 13 for more details†), the values of *s* can be extracted from the experimental data when the twist angle was 90° (see ESI note 7 for more details†). The experimental data were consistent with the theory (Fig. 4(f)). The calculations indicated that the behavior strongly depended on the thicknesses of BP and SiO_2_ layers.

**Fig. 5:**
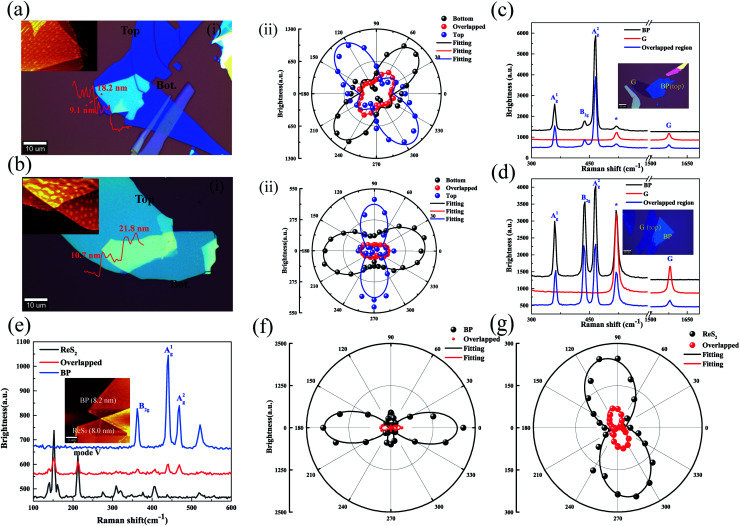
Polarization dependence of Raman modes for BP/BP, BP/ReS_2_, BP/G heterojunctions under the parallel polarization configuration on the 285 nm SiO_2_/Si substrate. (a and b) Polarization dependence of Raman modes for the BP/BP junction under parallel polarization configuration on the 285 nm SiO_2_/Si substrate. The thicknesses of the bottom BP were 9.1 and 10.7 nm. (i) The optical image of the BP/BP junction. (ii) A^2^_g_ mode as a function of the polarization angle for bottom BP, top BP, and the overlapped region. (c) The Raman spectrum of top BP, bottom G, and the overlapped region. The excitation wavelength of 532 nm was employed. Inset is the optical image of the BP/G heterojunction. (d) The Raman spectrum of bottom BP, top G, and the overlapped region. The excitation wavelength of 532 nm was employed. Inset is the optical image of the G/BP heterojunction. (e) The Raman spectrum of top ReS_2_, bottom BP, and the overlapped region. The excitation wavelength of 532 nm was employed. Inset is the AFM image of the BP/ReS_2_ heterojunction. (f) A^2^_g_ mode as a function of the polarization angle for BP. The excitation wavelength of 532 nm was employed. (g) V mode (213 cm^−1^) as a function of the polarization angle for ReS_2_. The excitation wavelength of 532 nm was employed.

Fig. 2(e) and 5(a(ii) and b(ii) show the polarization dependence of the A^2^_g_ mode for the BP/BP junction with different BP thicknesses under the parallel polarization configuration on the 285 nm SiO_2_/Si substrate. The values of *s* got smaller as the BP thicknesses increased from 7.3 to 10.7 nm. Because the ratio of the second maximum Raman intensity to maximum Raman intensity for the overlapped region got smaller, the values of *m* got larger. This was consistent with the simulation results (Fig. 1(b)).

The BP/ReS_2_ junction was assembled to demonstrate that the theory can be applied to the heterojunction assembled from different 2D materials. The Raman spectrum of the overlapped region showed no new peaks beyond the characteristic peaks of BP and ReS_2_ (Fig. 5(e)). Thus, the Raman signal in the overlapped region was indeed generated from the bottom and top samples, but with different interference factors.

Fig. 5(f and g) show the crystalline orientation of ReS_2_ and BP identified by the polarization dependence under the parallel configuration in the overlapped region. The ARPRS intensity of ReS_2_ and BP exhibits axisymmetry. The polarization dependence of each mode did not shift but Raman intensity decreased. Therefore, the polarization dependence of Raman modes for the overlapped region can be regarded as the sum of those generated by bottom and top samples with different *s*. *m* > 1 was observed for the overlapped region, which is consistent with the theory (Fig. 1(d)). Interference factors based on the multiple interference could help in clarifying the nature of such behaviors. Fig. 5(c and d) show the Raman spectrum of the BP/G heterostructure deposited on the 285 nm SiO_2_/Si substrate. The Raman intensity of the overlapped region was weaker than that of the independent region of BP and G, consistent with the theoretical predictions (Fig. 1(c)). It should be noted that our proposed multiple reflection interference was not unique to Raman measurements but can be applied to any spectroscopic measurements. Our work may benefit future studies related to Raman scattering of vdWHs.

**Fig. 6:**
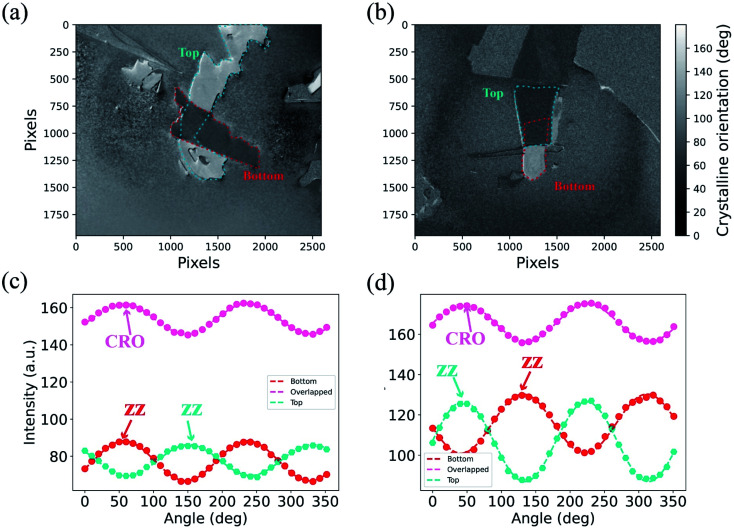
The crystalline orientation mapping for the BP/BP junction with a 90° twist angle on the 285 nm Si/SiO_2_ substrate. (a and b) The crystalline orientation mapping for the BP/BP junction on the Si substrates with 285 and 30 nm SiO_2_ layers. (c and d) The reflected light intensity spectra of top BP, bottom BP, and the overlapped region as a function of the rotation angle of the polarizer at a wavelength of 600 nm.

Angle-dependent reflection spectroscopy is one of the main methods to determine the crystalline orientation of layered 2D materials. AIT was used to investigate the crystalline orientation of BP and TBP. For the 285 nm SiO_2_/Si substrate, the CRO depended on the bottom BP (Fig. 6(a and c)), but at the 30 nm SiO_2_/Si substrate, the CRO obviously skewed toward the top BP (Fig. 6(b and d)). The experimental results showed that the reflection spectroscopy signal originating from the different layers can be modulated by the Si substrate with different SiO_2_ thicknesses. The experimental and theoretical results from the angle-dependent reflectance spectra with different BP thicknesses, wavelength, and the twist angle are shown in Fig. S5–S7,† which show that the wavelength and the twist angle caused a small impact in the relative reflection spectroscopy signal originating from different layers in vdWHs (see ESI for more details†). However, the thicknesses of the sample and the SiO_2_ layer can strongly influence the relative reflection spectroscopy signal that originated from different layers in vdWHs.

The Raman intensity of each vdWH mode was successfully studied. The values of *m* and *s* as a function of thicknesses of SiO_2_ and sample layers were studied both in theory and experiment. In general, the scale of *m* and *s* changed periodically with the thickness of the SiO_2_ layer. The zone of *s* < 1 was larger than that of *s* > 1 for the BP/BP junction, Thus, the maximum Raman signal of the overlapped region issued from bottom BP was larger than that induced by the top BP in a wide zone. As the sample thickness rose, *m* increased first and then decreased. In many cases, the zone observed for *m* > 1 was larger than that for *m* < 1. Therefore, the Raman intensity of the overlapped region was regularly weaker than that of the independent region and this was verified by studying the BP/BP, ReS_2_/BP, and G/BP vdWHS experimentally. The experiments showed that the Raman signal of the overlapped region on the 285 nm SiO_2_/Si substrate originating from bottom BP was larger than that induced by top BP. However, the Raman signal of the overlapped region on the 30 nm SiO_2_ layer originating from bottom BP was the same as that of top BP. Great agreement between the experimental data and the theory was observed when studying the BP/BP junction on the 30 nm Si/SiO_2_ substrate. The Raman intensity of each mode in vdWHs can be precisely predicted and modulated by the thicknesses of SiO_2_ and sample layers.

## 5. Conclusion

The Raman intensities of BP/G, BP/BP, and ReS_2_/BP vdWHs on a SiO_2_/Si substrate were all studied under parallel polarization, and we found that the contribution of the Raman signal induced by the underlying 2D material was much greater than that of the upper layer in some cases for the first time. The polarization dependence of Raman modes of BP/BP vdWHs was particularly studied by combining experiments with the theory. When two layers of nano-thick two-dimensional materials were stacked together, the ratio of interference factors for the bottom and top layers and the anisotropy of the heterojunction might be modulated by thicknesses of SiO_2_ and sample layers. In sum, the Raman intensity of each layer can precisely be modulated by its thickness. The results also demonstrated that the reflection spectroscopy signal generated by different layers in vdWHs can be modulated by the substrate, the sample thickness, and the twist angle. Interestingly, when the CRO depended on bottom layer 2D materials, the optical signal generated by the bottom layer 2D material was much larger than that of the top layer 2D material. Overall, these findings look promising for future studies of vdWHs.

## Conflicts of interest

The authors declare that they have no conflict of interest.

## Supplementary Material

NA-003-D0NA00906G-s001
