# Supplementary material for: Layer contribution to optical signals of van der Waals heterostructures
Source: Nanoscale Adv. 2021 Apr 10;3(11):3114–23. doi: 10.1039/d0na00906g (PMC9417842; doi:10.1039/d0na00906g)
Supplement: NA-003-D0NA00906G-s001 [file NA-003-D0NA00906G-s001.pdf]

Electronic Supplementary Material (ESI) for Nanoscale Advances.
This journal is © The Royal Society of Chemistry 2021

Supporting Information for

Layer contribution to optical signals of van der Waals heterostructures

Su-Yun Wang,† *Guo-Xing Chen,*† *Qin-Qin Guo,*† *Kai-Xuan Huang,*† *Xi-Lin Zhang,*† *Xiao-Qing Yan,*† *Zhi-Bo Liu,**,†,‡,§ *Jian-Guo Tian*†,‡,§

† The Key Laboratory of Weak Light Nonlinear Photonics, Ministry of Education, School of Physics and Teda Applied Physics Institute, Nankai University, Tianjin 300071, China

‡ Renewable Energy Conversion and Storage Center, Nankai University, Tianjin 300071, China

§ The collaborative Innovation Center of Extreme Optics, Shanxi University, Taiyuan, Shanxi 030006, China

* Correspondence to: liuzb@nankai.edu.cn

Supporting note 1: Interference factors calculation for BP/BP junction

Supporting note 2: Interference factors calculation with different N.A.

Supporting note 3: s>1 (the interference factor of the top BP is larger than that of the bottom BP)

Supporting note 4: Raman intensity of the top and bottom BP along AC and ZZ directions

Supporting note 5: The calculation of interference factors of the Raman signal for BP/BP junctions with a excitation wavelength of 514.4 nm

Supporting note 6: The calculation of interference factors of the Raman signal for BP/BP junctions with different refractive indexes.

Supporting note 7: The values of *s* extracted from the experimental data

Supporting note 8: BP/BP junction under the different wavelength

Supporting note 9: BP/BP junction with different twist angle

Supporting note 10: BP/BP junction with different thickness

Supporting note 11: BP/BP/BP three-layer junction

Supporting note 12: The Raman spectra of BP/BP junction under the parallel, cross and unpolarized configuration

Supporting note 13: Interference factors along with AC and ZZ direction of BP/BP junction

Supporting note 14: Refractive index used in the calculation

Supporting note 15: Reflected light intensity calculate

1. Interference factors calculation for BP/BP junction

For bottom BP layer (Fig. 1a(i)), the net absorption at position x can be expressed following Eq. (13)[1-3]:

$$F_{ab}^{bot}(x) = t_{02}\frac{\left(e^{-i\beta_x^{ex}} + r_{24}e^{-i\left(2\beta_2^{ex} - \beta_x^{ex}\right)}\right)}{\left(1 + \frac{\left(r_{12} + r_{01}e^{-2i\beta_1^{ex}}\right)}{\left(1 + r_{01}r_{12}e^{-2i\beta_1^{ex}}\right)}r_{24}e^{-2i\beta_2^{ex}}\right)} \quad (1)$$

$$t_{02} = \frac{t_{01}t_{12}e^{-i\beta_1^{ex}}}{\left(1 + r_{01}r_{12}e^{-2i\beta_1^{ex}}\right)} \quad (2)$$

$$r_{24} = \frac{\left(r_{23} + r_{34}e^{-2i\beta_3^{ex}}\right)}{\left(1 + r_{23}r_{34}e^{-2i\beta_3^{ex}}\right)} \quad (3)$$

where $r_{ij} = (n_i^{ex} - n_j^{ex})/(n_i^{ex} + n_j^{ex})$ and $t_{ij} = 2n_i^{ex}/(n_i^{ex} + n_j^{ex})$ are the reflectance and transmittance coefficients at interfaces of the i-th and j-th layer with i and j indices are given by air(0), top BP(1), bottom BP(2), SiO_2(3) and Si(4). n_i represents the complex refractive index of the i-th layer. The phase terms are $\beta_i^{ex} = 2\pi n_i^{ex}d_i/\lambda_{ex}$, $\beta_x^{ex} = 2\pi n_i^{ex}d_x/\lambda_{ex}$. d_i denotes the thickness of the i-th layer, λ_{ex} is the excitation wavelength.

The factors related to reflection of the Raman signal at position x can be expressed

by Eq. (16)[1-3]:

$$F_{sc}^{bot}(x) = t_{20}\frac{\left(e^{-i\beta_x^{sc}} + r_{24}e^{-i\left(2\beta_2^{sc} - \beta_x^{sc}\right)}\right)}{\left(1 + \frac{\left(r_{12} + r_{01}e^{-2i\beta_1^{ex}}\right)}{\left(1 + r_{01}r_{12}e^{-2i\beta_1^{ex}}\right)}r_{24}e^{-2i\beta_2^{sc}}\right)}\#() \quad 4.$$

$$t_{20} = \frac{t_{10}t_{21}e^{-i\beta_1^{sc}}}{\left(1 + r_{01}r_{12}e^{-2i\beta_1^{sc}}\right)}\#() \quad 5.$$

where $\beta_i^{sc} = 2\pi n_i^{sc}d_i/\lambda_{sc}$ and $\beta_x^{sc} = 2\pi n_i^{sc}d_x/\lambda_{sc}$ are the phase terms. λ_{sc} is the wavelength of the Raman signal, and n_i^{sc} represents the complex refractive index at the wavelength of the Raman signal.

The whole interference factors can be recalculated when considering both terms. Since the Raman intensity would be proportional to the enhancement factors ($F_{ab}^{bot}(x)$ and $F_{sc}^{bot}(x)$), so the total interference factors caused by the multiple interferences effect could be given by Eq. (18)[1-3]:

$$F_{bot} = N\int_0^{d_2}\left|F_{ab}^{bot}(x)\cdot F_{sc}^{bot}(x)\right|^2dx\#() \quad 6.$$

where N is a normalization factor.

For the top BP layer shown in Fig. 1a(ii), the net enhancement in excitation laser caused by multiple reflections at position x might be expressed by Eq. (19)[1-3]:

$$F_{ab}^{top} = t_{01}\frac{\left(e^{-i\beta_x^{ex}} + r_{14}e^{-i\left(2\beta_1^{ex} - \beta_x^{ex}\right)}\right)}{\left(1 + r_{01}r_xe^{-2i\beta_1^{ex}}\right)}\#() \quad 7.$$

$$r_{14} = \frac{\left[r_{12}\left(1 + r_{23}r_{34}e^{-2i\beta_3^{ex}}\right)e^{-2i\beta_2^{ex}} + \left(r_{23} + r_{34}e^{-2i\beta_3^{ex}}\right)e^{-2i\beta_2^{ex}}\right]}{\left[1 + r_{23}r_{34}e^{-2i\beta_3^{ex}} + r_{12}\left(r_{23} + r_{34}e^{-2i\beta_3^{ex}}\right)e^{-2i\beta_2^{ex}}\right]}\#() \quad 8.$$

Also, the net enhancement in scattered light might be given by Eq. (21)[1-3]:

$$F_{sc}^{top} = t_{10}\frac{\left(e^{-i\beta_x^{sc}} + r_{14}e^{-i\left(2\beta_1^{sc} - \beta_x^{sc}\right)}\right)}{\left(1 + r_{01}r_{14}e^{-2i\beta_1^{sc}}\right)} \quad 9.$$

The total interference factors of top BP layer can be expressed by Eq. (22)[1-3]:

$$F_{top} = N\int_0^{d_1} \left|F_{ab}^{top}(x) \cdot F_{sc}^{top}(x)\right|^2 dx \quad 10.$$

The analysis of the above-mentioned considerations revealed proportional relationships between the Raman intensities of the bottom or top BP layers and interference factors *F*.

2. Interference factors calculation with different N.A.

For s-polarized light, n_i could be replaced by $n_i cos(\theta_i)$. For p-polarized light, n_i could be replaced by $n_i/cos(\theta_i)$. $\beta_i = \frac{2\pi n_i d_i}{\lambda}$ could be replaced by $\beta_i = \frac{2\pi n_i d_i cos(\theta_i)}{\lambda}$. The weighting factor *g(θ)* reflects the Gaussian distribution of the incident laser.[1]

$$g(\theta) = \frac{9}{2\pi sin^2(\theta_{max})} e^{-\frac{1}{2}\left(\frac{3sin(\theta)}{sin(\theta_{max})}\right)^2}$$

where $\theta_{max} = asin(N.A./n_0)$. The total enhancement factors can be expressed as:

$$F = N\int_0^{\theta_{max}}\int_0^{d}\frac{1}{4}\sum_{j = s,p}\sum_{k = s,p}\left|F_{ab}^{j}(x,\theta)F_{ab}^{k}(x,\theta)\right|g(\theta)2\pi sin(\theta)cos(\theta)d\theta dx$$

3. s>1 (the interference factor of the top BP is larger than that of the bottom BP)

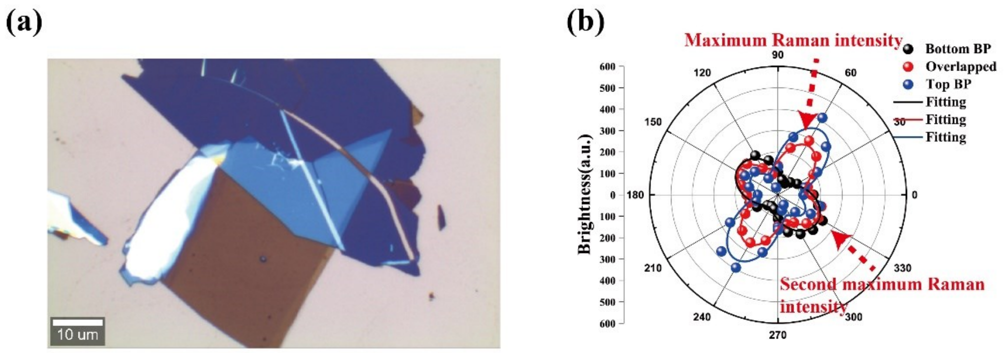

Fig. S1 Polarization dependence of Raman modes for BP/BP junction on the 30 nm SiO_2/Si substrate. (a) The optical image of the sample. (b) Polarization dependence of Raman modes for BP/BP junction under parallel polarization configuration on the 30 nm SiO_2/Si substrate.

We could easily find that the crystalline orientation of the overlapped region (CRO) is parallel to that of top BP instead of bottom BP, so s >1. s >1 exists in a small zone.

4. Raman intensity of the top and bottom BP along AC and ZZ directions

The Raman intensity of the top and bottom BP were compared. the ratio of Raman intensity in ZZ direction ($|I_{top}^{ZZ} - I_{bot.}^{ZZ}|/I_{top}^{ZZ}$) was 7.7%, and that in AC direction $|I_{top}^{AC} - I_{bot.}^{AC}|/I_{top}^{AC}$) was 8.4%. The errors are within 10%, and mainly caused by the processes of measurement and manufacture of TBP.

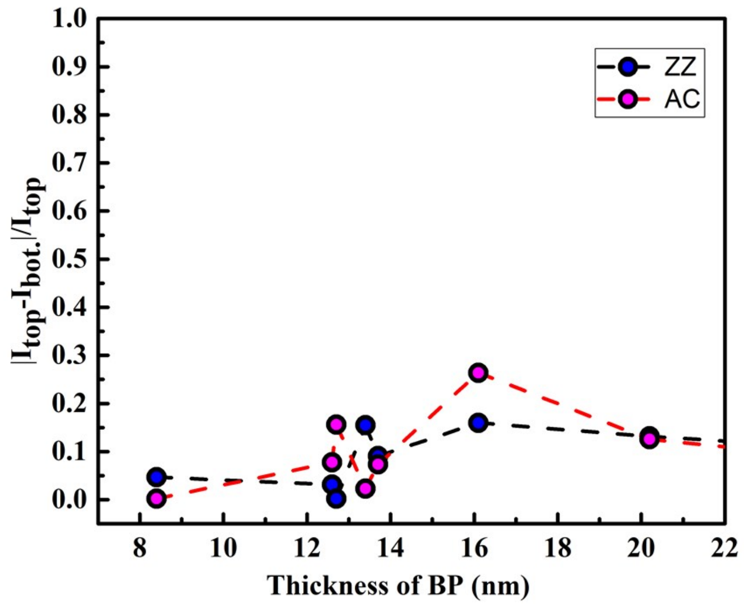

Fig. S2 The ratio of Raman intensity of the top and bottom BP along with AC and ZZ directions

5. The calculation of interference factors of the Raman signal for BP/BP junctions with a excitation wavelength of 514.4 nm

The variations in *m* and *s* as a function of the thicknesses of SiO_2 and BP layers for A_g^2 mode were illustrated in Fig. S3. Note that the thicknesses of the top and bottom BP were kept the same, and the excitation wavelength was 514.5 nm. The zone of *s* <1 can be visualized from Fig S3, which is wider than that of *s* >1. The zone of *m* >1 was much larger than that of *m* < 1 when the thickness of BP was less than 20 nm. The zones of s <1 and m >1 changed periodically with the thickness of SiO_2 layers and twist angle, but the latter demonstrated a smaller influence than the former.

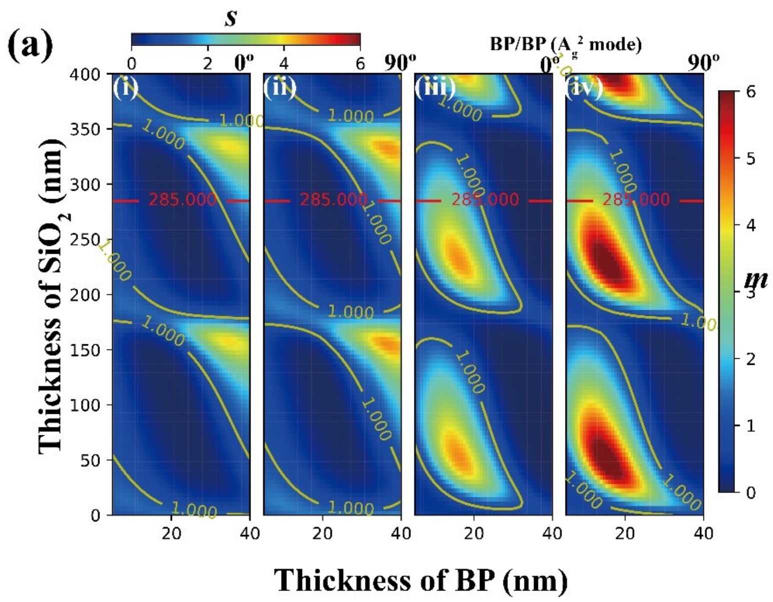

Fig. S3 The variations in *s* and *m* as a function of thicknesses of SiO_2 and BP layers. The excitation wavelength is 514.4 nm. The value of *s* was calculated and the stacking angle is (i) 0°, (ii) 90°. The value of *m* was calculated and the stacking angle is (iii) 0°, (iv) 90°.

6. The calculation of interference factors of the Raman signal for BP/BP junctions with different refractive indexes.

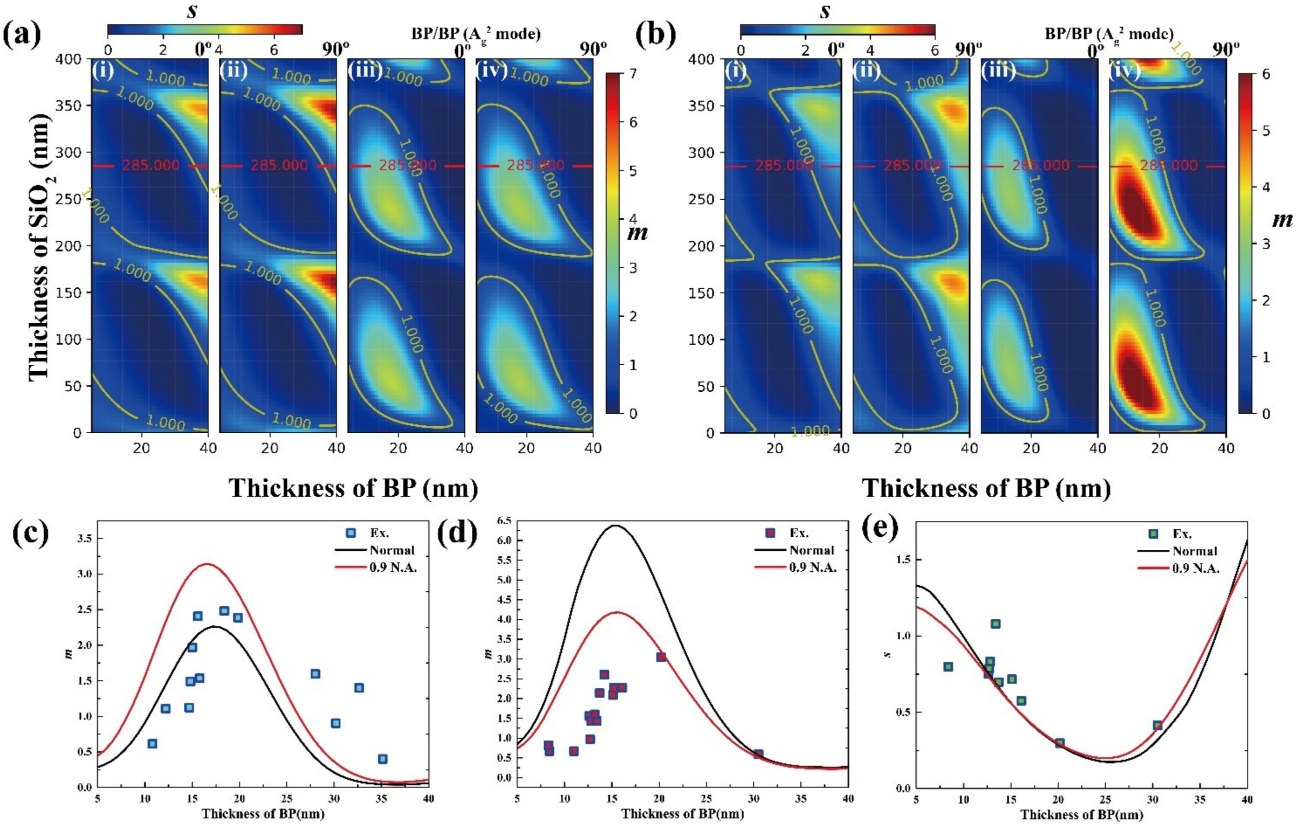

Fig. S4 The variations in s and m as a function of thicknesses of SiO_2 and BP layers. The value of s was calculated and the stacking angle is (i) 0°, (ii) 90°. The value of m was calculated and the stacking angle is (iii) 0°, (iv) 90°. (a) The refractive index used in reference [6]. (b) The refractive index used in reference [5]. (d, e) The theoretical and experimental data of the value of m (the ratio of the Raman intensity of bottom BP to that of the overlapped region) as a function of the thickness of BP/BP junction on the 30 nm SiO_2/Si substrate. The refractive index used in reference [5]. (f) The theoretical and experimental data of the value of s (the interference factors of bottom

BP to that of the top BP) as a function of the thickness of BP/BP junction on 30 nm SiO_2/Si substrate. The refractive index used in reference [5].

The different refractive indexes of BP were used in the calculation. It can be found that the zones of m>1 and s<1 show less different from each other. Therefore, the conclusion in this paper was almost the same when the different refractive indexes were used. Especially, the theoretical results using refractive in reference [5] was verified by the experiment. The values of m decreased slightly as the N.A. increased, and that of s almost kept the same. It is consistent with the experiment data when N.A. was considered. Therefore, the conclusion in this paper is supported by other literature quantitively.

7. The values of *s* extracted from the experimental data

Due to the interference factors between the ZZ and AC directions (F_{ZZ}/F_{AC}) was almost the same (~ 1.1). the values of *s* can be extracted from the experimental data when the twist angle was 90°, when s <1, the ratio between maximum Raman intensity and second maximum Raman intensity of the overlapped region can be expressed as eq. (1):

$$\frac{I_{max}^{ov.}}{I_{sec.}^{ov.}} = \frac{I_{max}^{bot.}F_b + I_{sec.}^{top}F_t}{I_{sec}^{bot.}F_b + I_{max}^{top}F_t} \#(1)$$

So, *s* can be expressed as eq. (2):

$$s = \frac{\left|\frac{a}{c}\right|^2 - \frac{I_{max}^{ov.}}{I_{sec.}^{ov.}}}{\left|\frac{a}{c}\right|^2 \cdot \frac{I_{max}^{ov.}}{I_{sec.}^{ov.}} - 1} \#(2)$$

where $\left|\frac{a}{c}\right|^2$ is ~0.3136.[3]

8. BP/BP junction under the different wavelength

The angle-dependent reflection spectra of the BP/BP junction with different wavelength were used to investigate how the twist angle influent the reflection spectroscopy signal generated by the bottom BP. As the wavelength increase from 500 to 600 nm, the CRO still parallel to the crystalline orientation of the bottom BP on the 285 nm SiO_2/Si substrate. It showed that the wavelength caused a small impact in the optical signal generated by different layers in vdWHs. At last, the experiment showed that the

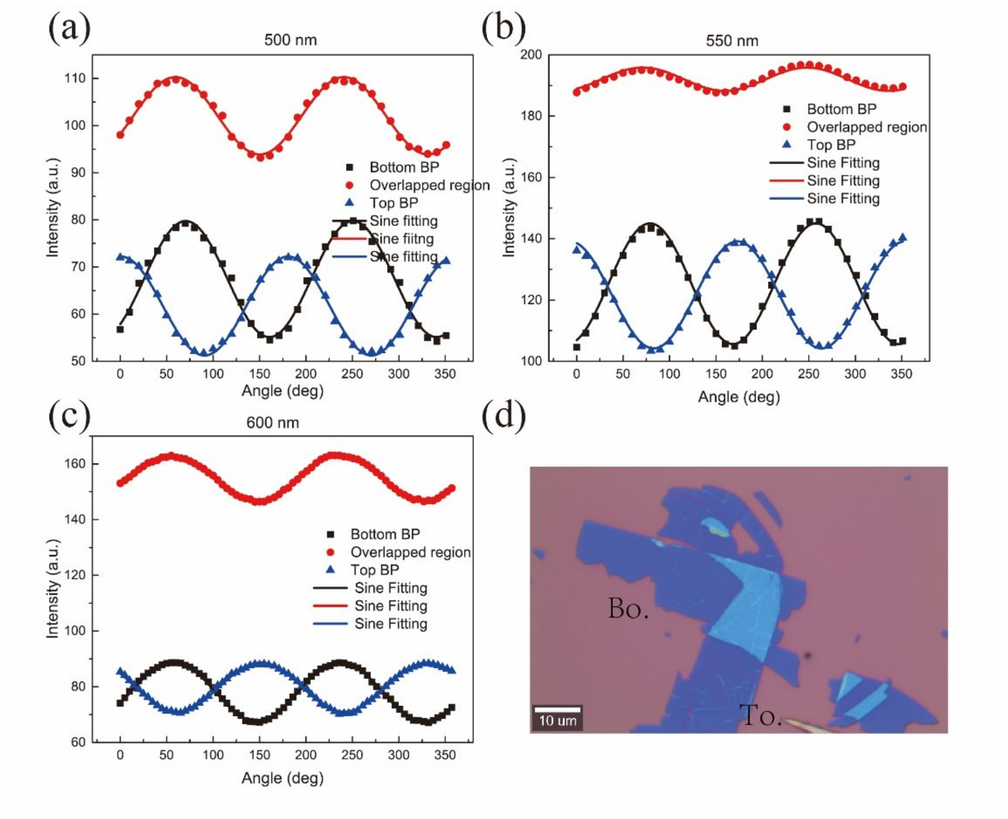

phenomenon is very normal.

Fig. S5 The reflected light intensity spectra of top BP, bottom BP, and the overlapped region as a function of the rotation angle of the polarizer with a 90° twist angle on the 285 nm SiO_2/Si substrate. (a) The wavelength is 500 nm. (b) The wavelength is 550 nm. (c) The wavelength is 600 nm. (d) The optical image of the sample.

9. BP/BP junction with different twist angle

The BP/BP junction with different twist angle was used to investigate how the twist angle influent the reflection spectroscopy signal generated by the bottom BP. At first, the thickness of the overlapped region is twice as thick as the independent BP region. the thickness of the independent BP is the same. Then, the CRO is parallel to the crystalline orientation of the bottom BP layer when the twist angle is 0° on the 285 nm SiO_2/Si substrate. As the twist angle of TBP increase, the CRO skewed toward the bottom BP, but the degree of it was reduced. Then, the experiment showed that the twist angel caused a small impact on the optical signal generated by different layers in vdWHs.

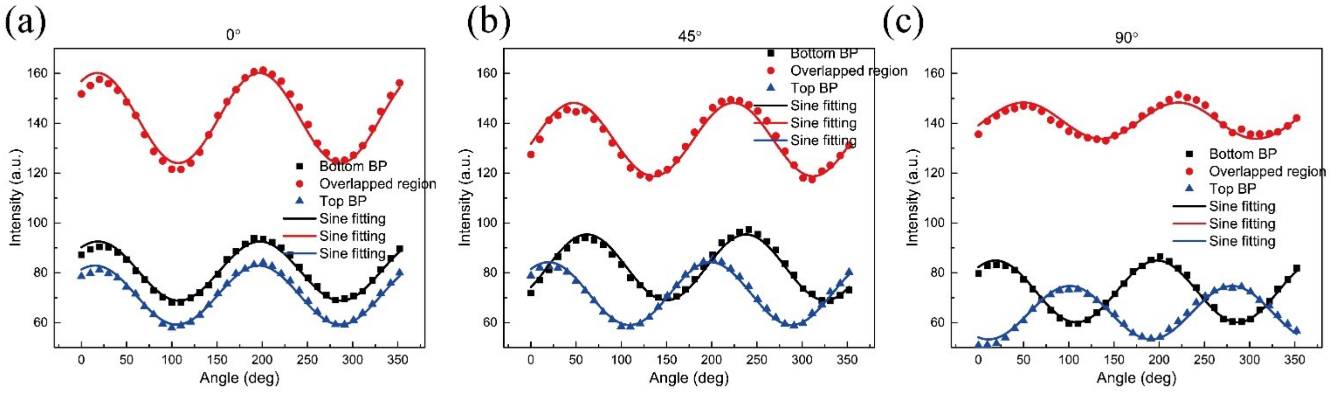

Fig. S6 The reflected light intensity spectra of top BP, bottom BP, and the overlapped region as a function of the rotation angle of the polarizer at a wavelength of 600 nm on the 285 nm SiO_2/Si substrate. (a) The twist angle is 0°. (b) The twist angle is 45°. (c) The twist angle is 90°.

10. BP/BP junction with different thickness

The BP/BP junction with different thicknesses was used to investigate how the thickness of the BP influent the reflection spectroscopy signal generated by the bottom BP. At first, the thickness of the overlapped region is twice as thick as the independent BP region. Then, the CRP is parallel to the crystalline orientation of the top BP layer when the thickness of the independent BP layer is 13.4 nm on the 30 nm SiO_2/Si substrate. But the CRO is parallel to the crystalline orientation of the bottom BP layer when the thickness of the independent BP layer is 17.1 nm on the 30 nm SiO_2/Si substrate. It showed that the thicknesses of the BP layer can modulate the reflection spectroscopy signal that originated from different layers in vdWHs.

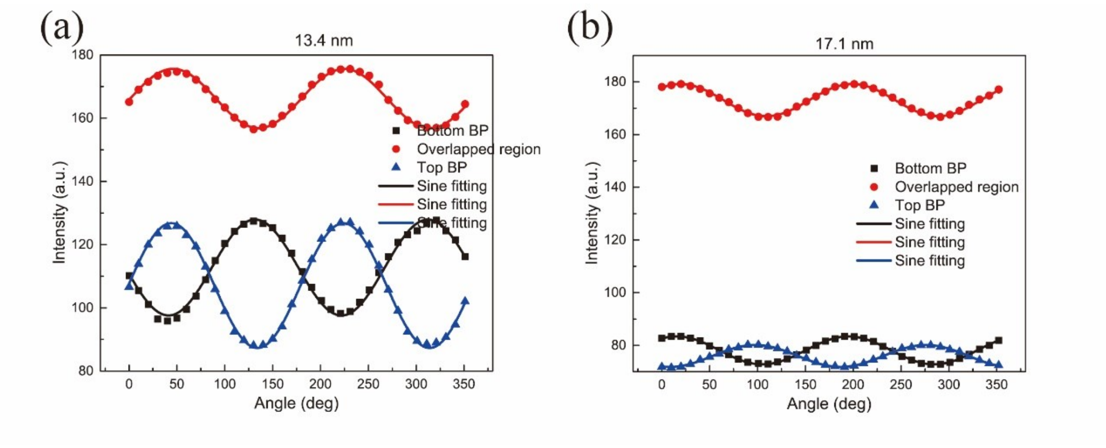

Fig. S7 The reflected light intensity spectra of top BP, bottom BP, and the overlapped region as a function of the rotation angle of the polarizer at a wavelength of 600 nm on the 30 nm SiO_2/Si substrate. (a) The thickness of the bottom BP is 13.4 nm. (b) The thickness of the bottom BP is 17.1 nm. The thickness of the bottom BP is the same as that of the top BP.

11. BP/BP/BP three-layer junction

The phenomenon is extremely obvious when the BP/BP/BP three-layer junction was synthesized. the Raman signal intensity of independent top BP layer is larger than bottom BP and smaller than middle BP layer. However, the crystalline orientation of overlapped region is skewed toward the bottom BP layer, which means that the Raman signal coming from bottom BP layer is larger than middle and top BP layer. the phenomenon is so abnormal and widely exists in the vdWHs. The theory and experiment have a certain contribution to the Raman spectrum of vdWHs.

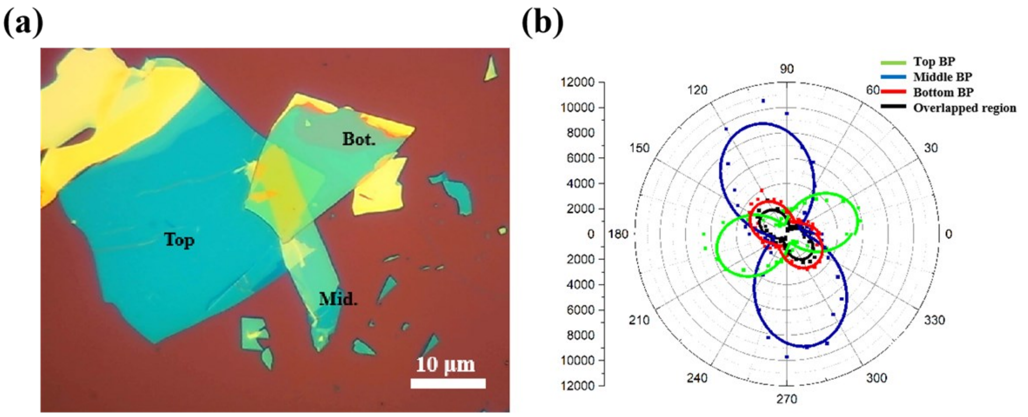

Fig. S8 Polarization dependence of Raman modes for BP/BP/BP junction on the 285 nm SiO_2/Si substrate. (a) The optical image of the BP/BP/BP junction. (b) A_g^2 mode as a function of polarized angle for the bottom BP, top BP, middle BP and overlapped region.

12. The Raman spectra of BP/BP junction under the parallel, cross and unpolarized configuration.

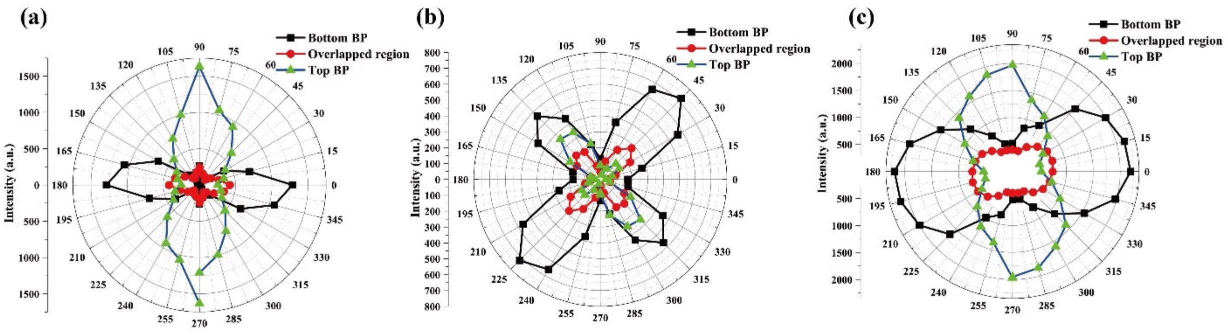

Fig. S9 (a), (b)Polarization diagram of the Raman intensity for A_g^2 mode under the parallel and cross configuration. (c) The Raman intensity of the BP/BP junction a for A_g^2 s a function of the polarized angle of 532 nm excitation laser.

The polarization direction of the incident laser is parallel (cross) to the polarization direction of the scattered light under the parallel (cross) configuration. The direction of the maximum Raman intensity for A_g^2 mode determined the crystalline orientation of the BP layer under parallel configuration. The changing curve showed the shape of a four-leaf clover under the cross configuration. The changing curve showed the sine shape under unpolarized configuration. The Raman spectra of the overlapped region were mainly dependent on that of the bottom BP under the unpolarized configuration.

13.Interference factors along with AC and ZZ direction of BP/BP junction.

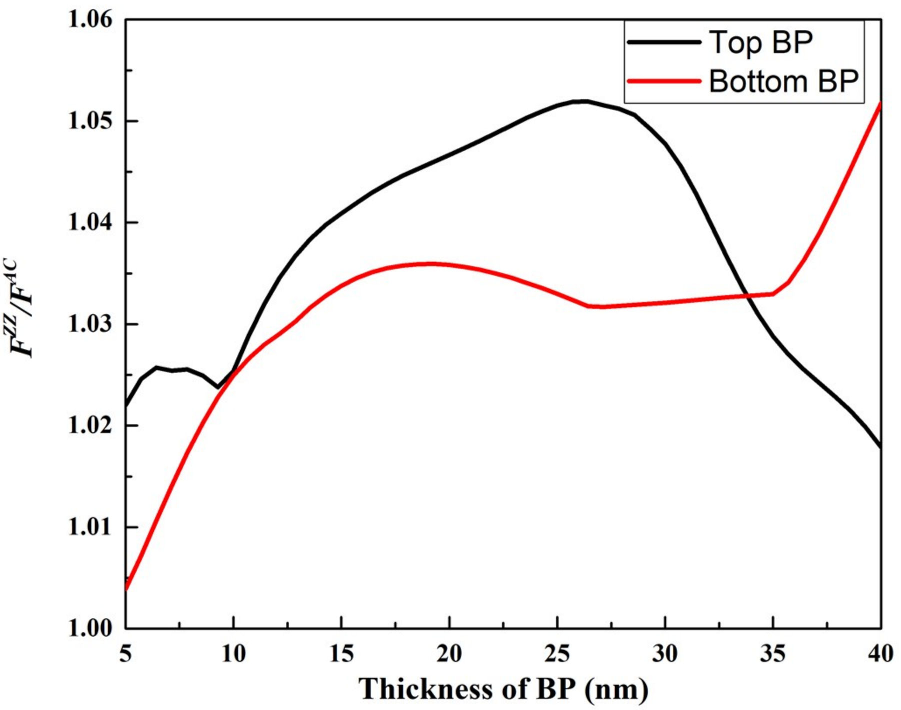

Fig. S10 The interference factors between the ZZ and AC directions (F^{ZZ}/F^{AC}) as a function of the thickness of BP/BP junction on the 30 nm SiO_2/Si substrate with 0.9 N.A..

The difference of the interference factors for 90° BP/BP junction along AC and ZZ directions can be reduced in contrast to 0° BP/BP junction. As Fig. S10 showed, the ratio of interference factors for 90° BP/BP junction between ZZ and AC directions (F^{ZZ}/F^{AC}) was ~1.03. therefore, the interference factors along with ZZ direction can be regarded as the same that along with AC direction on 30 nm SiO_2/Si substrate.

14. Refractive index used in the calculation

Table S1 Refractive index of BP used in the calculation.

**Wavelength (nm)**	**BP (zigzag)[6]**	**BP (armchair)[6]**	**SiO_2**	**Si[7]**
400	3.94-0.31i	3.56-0.12i	1.46	5.64-0.39i
532	3.55-0.96i	3.29-0.43i	1.46	4.12-0.08i;

The refractive index of BP at the wavelength of 514, 543 and 583 nm are calculated by the change trend.

Table S2 Refractive index of MoS_2, G, SiO_2, ReS_2, and Si.

**Wavelength (nm)**	**MoS_2[8]**	**G[9]**	**SiO_2**	**Si[7]**	**ReS_2[10] ($b_\perp$)**	**ReS_2[10] ($b_{\parallel}$)**
532	2.64-0.67i	2.68-1.22i	1.46	4.17-0.08i;	4.71-0.92i	4.91-1.27i
543	2.53-0.76i	2.68-1.24i	1.46	4.12-0.08i;	_	_
580	2.62-1.38i	2.69-1.29i	1.46	4.00-0.07i;	_	_

Table S3 Refractive index of BP in reference [4] and reference [5].

**Wavelength (nm)**	**BP (zigzag)[4]**	**BP (armchair)[4]**	**BP (zigzag)[5]**	**BP (armchair)[5]**
532	3.64-0.05i	3.57-0.37i	4.25-0.05i	4.10-0.55i
514.5	3.72-0.05i	3.68-0.38i	-	-

15. Reflected light intensity calculation

A five-phase model is presented to calculate the reflected light intensity, light is always normal incidence. *p* (electric-field vector E parallel to the incident plane) and *s* (electric-field vector E perpendicular to the incident plane) polarized light can be calculated independently. SO, *p* direction can be calculated before *s* direction. In this model, the whole system can be expressed by the transfer matrix:

$$S^p = I_{01}^p \cdot L_1^p \cdot I_{12}^p \cdot L_2^p \cdot I_{23}^p \cdot L_3^p \cdot I_{34}^p \quad \#(1)$$

In the transfer matrix, interface and interior should be expressed separately. The interfaces of different phase can be expressed by:

$$I_{m-1,m}^{\ p} = \frac{1}{t_{m-1,m}^{\ p}} \begin{bmatrix} 1 & r_{m-1,m}^{\ p} \\ r_{m-1,m}^{\ p} & 1 \end{bmatrix} \#(2)$$

Where $r_{m-1,m}$ is the reflection coefficient from layer *m-1* to *m*, and $t_{m-1,m}$ is the transmission coefficient from layer *m-1* to *m*.

When the light pass through the interior of different phase, the matrix is related to the optical path, so the matrix can be expressed by:

$$L_m^p = \begin{bmatrix} e^{i\delta_m^p} & 0 \\ 0 & e^{-i\delta_m^p} \end{bmatrix} \#(3)$$

Where δ_m^p is the phase shift induced by layer m along *p* direction.

Substitute the equation 2, 3 into the equation 1, the whole five-phase system can be expressed by:

$$\begin{bmatrix} E_{01}^+ \\ E_{01}^- \end{bmatrix} = \frac{1}{t_{01}t_{12}t_{23}t_{34}} \begin{bmatrix} 1 & r_{01} \\ r_{01} & 1 \end{bmatrix} \begin{bmatrix} e^{i\delta_{01}} & r_{12}e^{i\delta_{01}} \\ r_{12}e^{-i\delta_{01}} & e^{-i\delta_{01}} \end{bmatrix} \begin{bmatrix} e^{i\delta_{12}} & r_{23}e^{i\delta_{12}} \\ r_{23}e^{-i\delta_{12}} & e^{-i\delta_{12}} \end{bmatrix} \begin{bmatrix} e^{i\delta_{23}} & r_{34}e^{i\delta_{23}} \\ r_{34}e^{-i\delta_{23}} & e^{-i\delta_{23}} \end{bmatrix} \begin{bmatrix} 0 \\ E_{34}^- \end{bmatrix} \#(4)$$

Due to the normal incidence of the light, the reflection coefficient and transmission coefficient can be expressed by:

$$r_{m-1,m}^{p} = \frac{n_{m-1}^{p} - n_{m}^{p}}{n_{m-1}^{p} + n_{m}^{p}} \quad (5)$$

$$t_{m-1,m}^{p} = \frac{2n_{m-1}^{p}}{n_{m-1}^{p} + n_{m}^{p}} \quad (6)$$

$$\delta_{m}^{p} = \frac{2\pi d_m n_m^p}{\lambda} \quad (7)$$

Where d_m (m=1, 2, 3) is the thickness of layer m, the thickness of TBP is d_s, *np m* is the complex refractive index of layer m along p direction. n_0, n_3 and n_4 are the refractive index of air, SiO_2 and Si.[8] BP is biaxial crystal, whose refractive index is different along the three axes. Its two principal axes, ZZ and AC, are along p and s directions of bottom BP. So $n_2^p = n_{ZZ}$ and $n_2^s = n_{AC}$. Because the sample has the same thickness for top BP and bottom BP, the thickness of TBP can be regarded as two times thicker than BP $(d_1 = d_2, d_s = d_1 + d_2 = 2d_1)$. For the refractive index of top BP, we should do some approximation. In this approximation, the variation of n in the $p-s$ plane will form an elliptical shape (n along p direction n_p, n along s direction is n_s. n_{ZZ} and n_{AC} are the two axes of the ellipse (Fig. 4e). Therefore, along the given direction ϕ which is equivalent to p direction, $n(\phi) = n_1^p$ can be expressed as:[9]

$$n^2(\phi) = \frac{n_{AC}^2 n_{ZZ}^2}{n_{AC}^2 cos^2\phi + n_{ZZ}^2 sin^2\phi} \quad \#(8)$$

Where the twist angle is ϕ. The refractive index *np 1* is $n(\phi)$, and *ns 1* is $n(\phi+90°)$, Through the transfer matrix, we can get reflection coefficient and transmission coefficient of the whole system, and the reflectance coefficient and transmission coefficient of this system can be expressed by:

$$r^{p(s)} = \frac{E_{01}^{-}}{E_{01}^{+}} = \frac{S_{2,1}^{p(s)}}{S_{1,1}^{p(s)}} \#(9)$$

$$t^{p(s)} = \frac{E_{34}^{-}}{E_{01}^{+}} = \frac{1}{S_{1,1}^{p(s)}} \quad \#(10)$$

the reflected light intensity can be expressed by:

$$R^{p(s)} = r^{p(s)} \cdot r^{p(s)\,*} \#(11)$$

Supplementary References

1. D. Yoon, H. Moon, Y. W. Son, J. S. Choi, B. H. Park, Y. H. Cha, Y. D. Kim and H. Cheong, Phys. Rev. B, 2009, **80**, 125422

2. X. Ling, S. Huang, E. H. Hasdeo, L. Liang, W. M. Parkin, Y. Tatsumi, A. R. T. Nugraha, A. A. Puretzky, P. M. Das, B. G. Sumpter, D. B. Geohegan, J. Kong, R. Saito, M. Drndic, V. Meunier and M. S. Dresselhaus, Nano Lett., 2016, **16,** 2260-2267

3. J. Kim, J. U. Lee, J. Lee, H. J. Park, Z. Lee, C. Lee and H. Cheong, Nanoscale, 2015, **7**, 18708-18715

4. H. Asahina and a. Morita, J. Phys. C: Solid State Phys., 1984, **17**, 1839 —1852

5. M. L. Lin, Y. C. Leng, X. Cong, D. Meng, J. Wang, X. L. Li, B. L. Yu, X. L. Liu, X. F. Yu and P. H. Tan, Science Bulletin, 2020, **65**, 1894-1900

6. H. Jiang, H. Shi, X. Sun and B. Gao, ACS Photonics, 2018, **5**, 2509-15

7. J. Šik, J. Hora and J. Humlíček, J. Appl. Phys., 1998, **84**, 6291-6298

8. C. Yim, M. O'Brien, N. McEvoy, S. Winters, I. Mirza, J. G. Lunney and G. S. Duesberg, Appl. Phys. Lett., 2014, **104**, 103114

9. J. W. Weber, V. E. Calado and M. C. M. van de Sanden, Appl. Phys. Lett., 2010, **97** 09190

10. H. Jiang, H. Shi, X. Sun and B. Gao, Applied Physics Letters, 2018, ***113***, 213105.
